# Viromics Reveal a Number of Novel RNA Viruses in Swedish Mosquitoes

**DOI:** 10.3390/v11111027

**Published:** 2019-11-05

**Authors:** Pontus Öhlund, Juliette Hayer, Hanna Lundén, Jenny C. Hesson, Anne-Lie Blomström

**Affiliations:** 1 Department of Biomedical Sciences and Veterinary Public Health, Swedish University of Agricultural Sciences, Box 7028, 750 07 Uppsala, Sweden; Hanna.lunden@slu.se (H.L.); anne-lie.blomstrom@slu.se (A.-L.B.); 2 Department of Animal Breeding and Genetics, Swedish University of Agricultural Sciences, SLU-Global Bioinformatics Centre, Box 7023, 750 07 Uppsala, Sweden; juliette.hayer@slu.se; 3 Department of Medical Biochemistry and Microbiology/Zoonosis Science Center, Uppsala University, Box 582, 751 23 Uppsala, Sweden; jenny.hesson@imbim.uu.se

**Keywords:** insect-specific virus, metagenomics, virome, mosquitoes

## Abstract

Metagenomic studies of mosquitoes have revealed that their virome is far more diverse and includes many more viruses than just the pathogenic arboviruses vectored by mosquitoes. In this study, the virome of 953 female mosquitoes collected in the summer of 2017, representing six mosquito species from two geographic locations in Mid-Eastern Sweden, were characterized. In addition, the near-complete genome of nine RNA viruses were characterized and phylogenetically analysed. These viruses showed association to the viral orders *Bunyavirales*, *Picornavirales*, *Articulavirales*, and *Tymovirales*, and to the realm Ribovira. Hence, through this study, we expand the knowledge of the virome composition of different mosquito species in Sweden. In addition, by providing viral reference genomes from wider geographic regions and different mosquito species, future in silico recognition and assembly of viral genomes in metagenomic datasets will be facilitated.

## 1. Introduction

Mosquitoes are known to host and transmit numerous arthropod-borne viruses (arboviruses) that cause disease both in humans and animals, such as West Nile virus (WNV) [1], Zika virus [2], chikungunya virus [3], and dengue virus [4]. However, mosquitoes can harbour more than pathogenic arboviruses. Large metagenomic surveys of invertebrates, enabled via the use of high-throughput sequencing and advanced bioinformatics, have led to the discovery of numerous novel viruses [5,6,7,8,9,10]. Hence, these studies have shown that the mosquito virome is highly diverse and includes viruses restricted to arthropods, more commonly known as insect-specific viruses (ISVs). Although these viruses are harmless to humans and animals and have no direct impact on public health, they are interesting for multiple reasons. Several studies have shown that ISVs could potentially alter the mosquito’s susceptibility to carry and transmit pathogens of concern for animal and human health [11,12,13,14,15,16]. Furthermore, some of the ISVs belong to viral families associated with arboviruses such as *Peribunyaviridae*, *Flaviviridae*, *Reoviridae,* and *Togaviridae* and are thought to be ancestral to arboviruses. With this in mind, ISVs could be a potential source of new arboviruses if they acquire dual-host tropism [7,17,18]. Therefore, by studying the virome of mosquitoes from different regions and from different mosquito species, we will not only have the opportunity to discover new viruses but can also learn more about viral evolution and the factors influencing vector competence.

The majority of the metagenomic surveys of mosquitoes have been conducted in countries where arboviruses of human and animal health concern circulate, such as China, Australia, Mozambique, and the US [5,6,19,20]. In Sweden, two mosquito-borne viruses circulate: the arthralgia-associated Sindbis virus (SINV) [21] and Inkoo virus (INKV), which in rare cases, may cause mild encephalitis [22,23]. Further, it has been discussed whether WNV can be introduced to Sweden as it circulates in Southern and Central Europe, and the mosquito vector species, such as *Culex (Cx.) pipiens* and *Cx. Torrentium*, are already present in the country [24,25].

In this study, we aimed to provide additional knowledge of viruses circulating in Northern Europe, as well as investigate the virome composition of different mosquito species in this region by using a viral metagenomic approach. In total, the virome composition of six different mosquito species collected in two locations in Mid-Eastern Sweden were investigated. In addition, we have further genetically characterised nine near-complete viral genomes associated with the orders *Bunyavirales*, *Picornavirales*, *Articulavirales*, and *Tymovirales*, and the realm Ribovira. 

## 2. Materials and Methods

### 2.1. Mosquito Collection, Species Separation and Pool Design

Mosquitoes were collected in the summer of 2017 at two different geographic locations in Mid-Eastern Sweden, close to the small town Flen (coordinate: 59°04′08.6″ N 16°31′14.1″ E) and in the nature reserve Hammarskog (coordinate: 59°46′31.9″ N 17°35′01.3″ E) close to the city of Uppsala. In the location close to Flen, mosquitoes were collected once every second week from May to the end of August, using dry-ice-baited Centers for Disease Control and Prevention (CDC) miniature light traps and CDC gravid traps (John W. Hock company, Gainesville, FL, USA). In Hammarskog, a Mosquito Magnet^®^ was placed close to a bird breeding ground from Monday to Friday every second week, from July to the end of August. The Mosquito Magnet^®^ was emptied every 24 h. The mosquitoes were euthanized and stored at −80 °C after collection. Female mosquitoes were determined to species based on morphological character, using a taxonomic key [26], stereomicroscope, and a cold table. *Culex* (*Cx*) torrentium and *Cx*. pipiens were identified to species using a molecular method previously described [27]. Eight to twelve female mosquitoes were pooled based on mosquito species, time of collection, and location. The most abundant species collected (*Coquillettidia* (*Cq) richardii*, *Aedes (Ae) communis*, *Ae*. *annulipes*, *Ae. cantans* and all *Cx. pipiens and Cx. torrentium)* were used in further analyses.

### 2.2. Homogenisation, Nucleic acid Extraction, Pre-Sequencing Preparation and Sequencing

Prior to homogenisation, all mosquitoes were washed once in 70% ethanol and then twice in milliQ water to remove potential surface microorganisms. The mosquitoes in each pool were then transferred to Precellys tubes (soft tissue homogenization CK14) and 700 μL PBS supplemented with Amphoterecin (250 μg/mL) and Penicillin-Streptomycin (100 U penicillin/mL + 100 μg streptomycin/mL) was added. Homogenisation was performed mechanically with a Precellys^®^ evolution (Bertin instruments, Paris, France) at two cycles of 5500 RPM for 30 s with a 40 s pause between each cycle. In addition, to keep the temperature at 10 °C a Cryolys (Bertin instruments, Paris, France) cooled with liquid nitrogen was coupled to the Precellys^®^ evolution. The homogenates were centrifuged at 8000× *g* for 5 min at 4 °C and the supernatant was collected for future analysis. Total RNA was extracted with TRIzol™ according to the manufacture’s protocol. The aqueous phase obtained after the addition of chloroform and the subsequent centrifugation step was collected and diluted 1:1 with freshly prepared 70% ethanol and purified with GeneJet spin columns. The purified RNA was further pooled with RNA from the same mosquito species, location, and time point, resulting in a total of 12 pools (Table 1). These pools were subjected to DNAse treatment using RNAeasy mini elute kit and RNase-Free DNase set (Qiagen, Hilden, Germany), following the manufacturer’s recommended protocol. Ribosomal RNA was removed using the Ribo-Zero Gold (epidemiology) rRNA Removal Kit (Illumina, San Diego, USA) and the non-ribosomal RNA was randomly amplified with the Ovation^®^ RNA-Seq System V2 (NuGEN Technology, San Carlos, CA, USA), following the manufacture’s instruction. The amplified RNA was submitted to SciLifeLab for library preparation and sequencing using the Ion 530™ Chip kit (Thermo Fisher scientific, Massachusetts, USA). The 12 pools were dived on four Ion 530™ chips and sequencing was carried out with an Ion S5 XL sequencing system running the protocol for 400 bp-long reads.

### 2.3. Metagenomics Data Analysis

The Ion S5 XL sequence data were analysed in a metagenomics pipeline starting with quality control using FastQC (v1.2.1). Reads of low quality were trimmed with sickle (version 1.210) using a cut-off PHRED score of 20. The mean reads length after trimming was 359–380 nt. The good quality reads were de-novo assembled using MEGAHIT (v1.1.2). Both the good quality reads and the de-novo assembled contigs were taxonomically assigned in separate runs using Diamond version 0.9.10 with the blastx option using the default settings (e-value cutoff 0.001 and one top hit) and the NCBI database from October 2018. In order to visualize the results with the R-package Pavian (v0.8.4), the output format taxonomy (102) was used in Diamond. This option performs a LCA (Last Common Ancestor) algorithm to assign the taxonomy of each read and output a tabulated file (https://github.com/bbuchfink/diamond/blob/master/diamond_manual.pdf). For the de-novo assembled contigs, an additional Diamond run was performed to output a DAA (Direct Access Archive) format that could be imported into MEGAN6 (version 6.15.2) for further investigation. Potential viral sequences were analysed with NCBI’s ORF finder (https://www.ncbi.nlm.nih.gov/orffinder/), functional annotation of the ORFs was performed using NCBI’s BLASTp, EMBL-EBI’s protein sequence analysis and classification tool InterProScan [28] and Phobius [29]. Phylogenetic analyses were performed on the RNA-dependent RNA polymerase (RdRp) amino acid sequences for the novel viruses and related sequences in the GenBank database with >20% amino acid (aa) identity. Sequence alignment and phylogenetic tree building were performed in MEGA 7 [30]. MUSCLE was used for multiple sequence alignment of the amino acid (aa) sequences [31]. The phylogenetic trees were computed with the maximum-likelihood method using 500 bootstraps replicates.

### 2.4. PCR Gap Closure of Viral Genome

We further investigated the Hammarskog picorna-like virus (HPLV) with PCR and Sanger sequencing. Viral hits were inspected and retrieved in Megan, overlapping contigs that mapped to the same virus were manually assembled into longer sequences. The extended sequences were subsequently used to design primers pairs to fill gaps and cover as much as possible of the genome using the primer3 software. The original RNA extractions of interest were reverse transcribed to cDNA using SuperScript^®^ III (Invitrogen, Carlsbad CA, USA). PCR reactions were carried out with KAPA2G Robust Hotstart ReadyMix (KAPABiosystems, Wilmington, MA, USA). PCR products were analysed with electrophoresis and bands of correct size were cut out and extracted with a GenJet gel extraction kit (Thermo Fisher Scientific, Waltham, MA USA). Purified PCR products were sent to Macrogen for Sanger sequencing and the returning sequences were analysed in DNASTAR, SeqMan Pro (Version 2.1.0.97).

### 2.5. Accession Numbers

The raw sequence reads generated in this study are available at the NCBI sequence read archive (SRA) database under Bioproject accession (PRJNA574583). All virus genome sequences retrieved in this study have been deposited in GenBank under the accession numbers: MN513369–MN513382.

## 3. Results

### 3.1. Mosquito Collection

**Figure 1:**
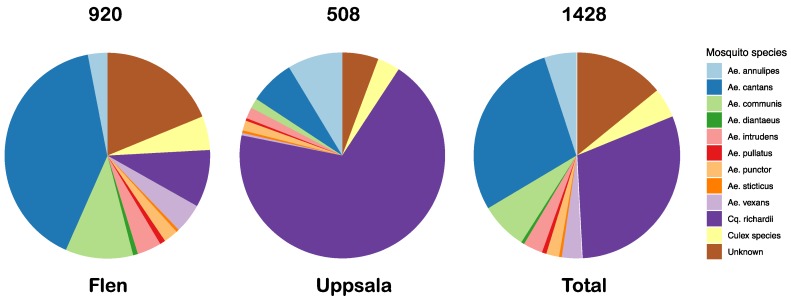
Distribution of all mosquito species collected in Flen and Uppsala. Mosquito species are represented by different colors. *Aedes (Ae), Coquillettidia* (*Cq*).

In total, 1428 mosquitoes of at least 12 different species were collected at the two different locations; 920 mosquitoes in Flen and 508 mosquitoes in Uppsala. The most abundant mosquito species were *Cq. richardii* (433), *Ae. cantans* (407), *Ae. communis* (106) and *Ae. annulipes* (72). The less abundant mosquito species out of those collected were *Ae. vexans* (45), *Ae. intrudens* (44), *Ae. punctor* (29), *Cx. pipiens* (22) and *Cx. torrentium* (4) (Figure 1). 

### 3.2. The Mosquito Viromes

**Figure 2:**
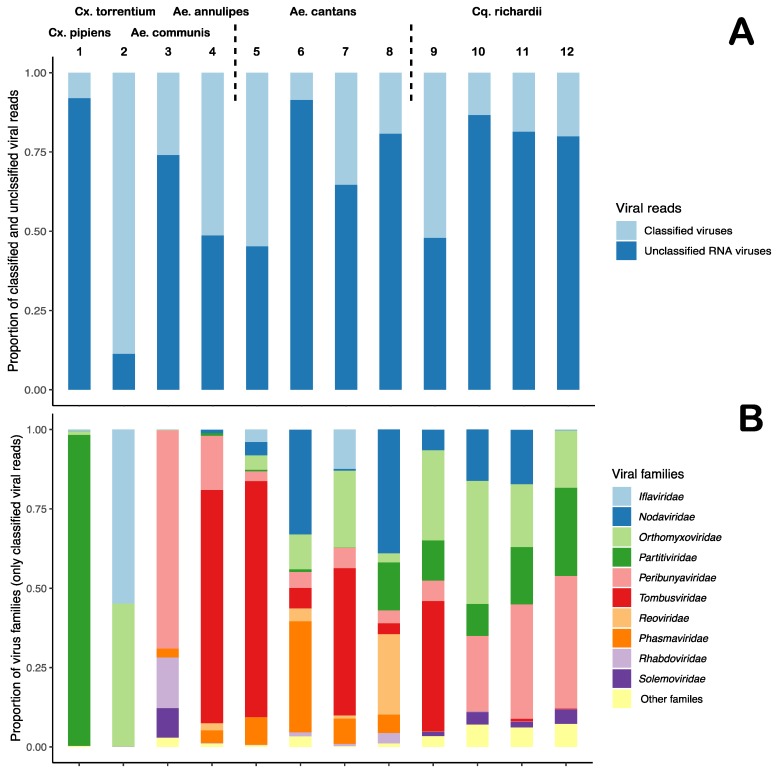
Annotation of viral reads in the different pools of mosquitoes. Each bar represents a mosquito pool—designated 1–12 and are described in Table 1. (**A**) Proportion of classified viral reads and unclassified RNA viral read. (**B**) Proportion of virus families of the classified viral reads.

We have characterized the RNA viromes of 953 female mosquitoes, divided into 12 pools, representing six mosquito species from two geographic locations in Mid-Eastern Sweden. For each mosquito pool, between 1.4–9.2 million reads were obtained, of these 1.4–42.6% mapped to diptera and 13.2–56.2% were microbial reads, of which 1.44–12.5% of the reads were classified as viral (Table 1). A large proportion of the viral reads were labelled as unclassified RNA viruses (Figure 2A). In the unclassified virus group, the majority of the viral sequences showed close to distant similarity (28–96% amino acids (aa) identity) to various viruses previously identified through different large metagenomic surveys of invertebrates [5,6,7]. The classified viral reads belonged to the families *Iflaviridae*, *Nodaviridae*, *Orthomyxoviridae*, *Partitviridae*, *Peribunyaviridae*, *Phasmaviridae*, *Reoviridae*, *Rhabdoviridae*, *Solemoviridae,* and *Tombusviridae* (Figure 2B). De novo assembly of the viral reads produced the near-complete genomes of nine viruses in the orders *Picornavirales*, *Tymovirales*, *Bunyavirales*, and *Articulavirales*, and the realm Riboviria.

### 3.3. Diverse Distribution of Viral Reads

The viromes analysed in this study were diverse and many viral families were represented (Figure 1 and Table 2). Differences between mosquito species and the types of viruses they harboured were observed, however, a large number of viral hits were observed across more than one species and genera. For example, viral reads that mapped to the Culex mononega-like virus 2 (CMLV2) were observed in all pools except in the *Cx. pipiens* mosquitoes and in one pool of *Ae. Canatans*, collected late in the season. Reads that mapped to CMLV2 were particularly abundant in the *Cx. torrentium* (0.216% total reads (TR)), *Ae. communis* (0.112% TR) and *Ae. annulipes* mosquitoes (0.721% TR) (Table 2). In terms of mosquito genera, many similarities could be observed. For example, the *Aedes* mosquitoes had many common negative-sense RNA viral hits, such as those showing similarity to Yongsan bunyavirus 1 (YBV1) (0.016–2.844% TR), Xincheng anphevirus (0.004–0.142% TR), Anopheles darlingi virus (0.001–0.381% TR), and Wuhan mosquito orthophasmavirus 2 (0.003–0.215% TR) (Table 2). However, the *Culex* mosquito species only shared one viral hit, the Culex Iflavi-like virus 3, which was exceptionally abundant in the *Cx. torrentium* (2.3% TR) compared to the *Cx. pipiens* (0.003% TR). Many of the viral hits were restricted to one mosquito species, such as similarity hits to the Hubei virga-like virus 21, which was only observed in the *Cx. pipiens* mosquitoes and in those, represented more than half of the viral reads in that pool (4.916% TR) (Table 2). Moreover, several of the unclassified RNA virus hits were only observed in the *Cq. richardii* pools, such as, Hubei noda-like virus 12 (0.023–4.22% TR), Hubei diptera virus 13 (0.022–0.074% TR), Hubei sobemo-like virus 8 and 9 (0.02—0.064%, 0.022–0.069% TR) and the Hubei tetragnatha maxillosa virus 8 (0.015–0.48% TR) (Table 2). Viral reads showing similarity to the positive-sense RNA virus Yongsan picorna-like virus 3 (YPLV3) were also only observed in the *Cq. richardii* pools, with high abundance in those collected in Uppsala (0.124–1.279% TR) and lower abundance in those collected in Flen (0.002% TR) (Table 2). The *Ae. cantans* mosquitoes shared most of its viral hits with other mosquito species, except four viruses; two positive-sense RNA viruses, Wuhan mosquito virus 3 (0.006–0.119% TR) and the Whidbey virus (0.007–0.102% TR) and two negative-sense RNA viruses, Kinkell virus (0.087–0.109% TR) and the unclassified virus Shuangao insect virus 12 (0.024–0.166% TR). Comparing the virus composition and abundance between the species and genera in this dataset, one can observe some differences. The *Aedes* genus seems to harbour a more diverse negative-sense RNA virome compared to the *Culex* and *Coquillettidia* genera. The *Culex* mosquitoes had few but very abundant viruses, one that represented more than half of the total viral reads in the *Cx. pipiens* pool. Many viral hits could only be observed in one of the mosquito species, indicating a host restriction. No significant differences could be observed comparing the locations or time point of collection in the *Cq. richardii* pools and *Ae. cantans* pool (Table 2). 

### 3.4. Positive-Sense RNA Viruses

The positive-sense RNA viruses observed in our mosquitoes were within the viral familes/orders *Iflaviridae*, *Nodaviridae*, *Tombusviridae*, *Solemoviridae*, and *Picornavirales* and represented between 0.03–47% of the viral reads in the mosquito pools. Four of the positive-sense RNA viruses detected were further characterized and phylogenetically analysed and fell within the viral orders *Picornavirales* and *Tymovirales.*

**Figure 3:**
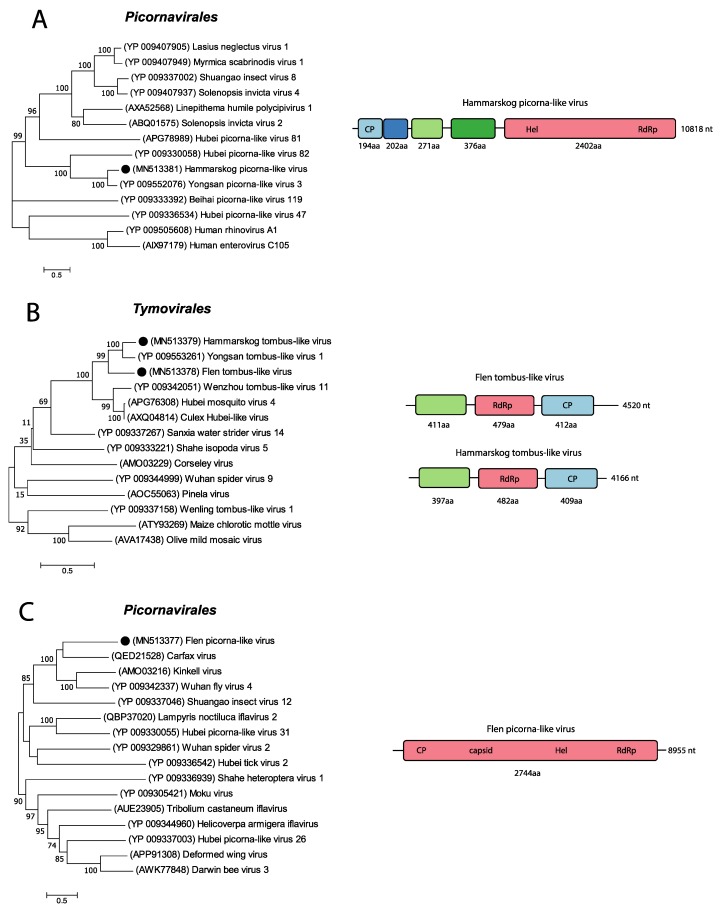
Phylogenetic analysis and genomic features of the positive-sense RNA viruses discovered in this study. The maximum-likelihood phylogenetic trees show the positions of newly discovered viruses (solid black circles) in the context of representatives of their closest relatives. The genome structures of viruses discovered in this study are shown next to their corresponding phylogenies. (**A**) The phylogenetic tree was generated using the translation of the fifth ORF. (**B**) Phylogenetic tree was generated using the translation of the second ORF for both FTLV and HTLV. (**C**) The phylogenetic tree was generated using the aa sequence of the complete polyprotein. RNA-dependent RNA polymerase (RdRp).

The analysed high-throughput data from the three pools of *Cq. richardii* mosquitoes collected in Uppsala showed many contigs that mapped to the Yongsan picorna-like virus 3 (YPLV3) (NC_040584.1). Using these contigs, combined with PCR and Sanger sequencing, the near-complete genome of a picorna-like virus, that we named “Hammarskog picorna-like virus” (HPLV) (Figure 3A), was recovered. This near-complete genome was 10,818 nt long and the sequence was further confirmed by mapping raw reads from the respective pools to the retrieved sequence. BLASTn analysis confirmed that the YPLV3 (NC_040584.1) had the closest similarity to the picorna-like sequence identified in this study with a nucleotide (nt) identity of 69.71%. YPLV3 was discovered in *Ae. vexans nipponii* mosquitoes collected in the Republic of Korea [32] and has a genome of 11,291 nt with four described ORFs coding for three small hypothetical proteins and a polyprotein. Analysing the HPLV genome with NCBI’s ORF finder software resulted in five ORFs, i.e., one extra ORF of 202 aa was identified between the first and the second ORF described by Sanborn et al. [32]. However, analysing the YPLV3 (NC_040584.1) genome with NCBI’s ORF finder showed that it also contained this additional ORF (191 aa). Sequence comparison of these two ORF sequences showed that they share a 62% aa identity to each other. No function of the protein could be predicted. Furthermore, the first ORF of our sequence translated to a protein of 194 aa and is predicted to code for a viral coat protein subunit (IPR029053). The third and fourth ORF code for proteins of 271 aa and 376 aa with no predicted functions. The fifth ORF had no stop codon, which suggests that our sequence is incomplete, in addition to lacking a 3’ UTR. Moreover, the fifth ORF is predicted to code for a polyprotein of 2402 aa that would be processed into a peptidase S1 (IPR009003), Helicase super family 3 (IPR000605, IPR014759) and an RNA-directed RNA polymerase (RdRp) (IPR001205) from the N- to C-terminus direction. BLASTp analyses of HPLV’s hypothetical proteins showed an aa identity of 58.55%, 74.17%, 60.95%, and 66.54% to each respective hypothetical protein of YPLV3. Phylogenetic analysis of the polyprotein and related sequences showed that our sequence clustered with picorna-like viruses discovered in insects, such as the Hubei picorna-like virus 82 (YP_009330058) (Figure 3A). 

Further, two near-complete genomes showing 67.63% and 75.17% nt identity to the Yongsan tombus-like virus 1 (YTLV1) (NC_040725) were identified. One of the genomes was found in the *Ae. cantans* collected late in the season in Flen and one genome was found in the *Cq. richardii* mosquitoes collected early in the season in Uppsala. The tombus-like viral contig retrieved from *Ae. cantans* mosquitoes was 4520 nt long and the virus was named Flen tombus-like virus (FTLV), while the tombus-like viral contig retrieved from the *Cq. richardii* mosquitoes was 4166 nt long and this virus was named Hammarskog tombus-like virus (HTLV) (Figure 3B). Sequence comparison between FTVL and HTLV showed that they are different tombus-like viruses as they only showed an nt identity of 64.5% to each other. Using NCBI’s ORF finder, it was predicted that both genomes had three ORFs of similar size and position as the YTLV1 (Table 3). 

The InterProScan analysis of each ORF’s aa sequences revealed no predicted function for the first ORF, the second ORF showed a detailed signature match of a RdRp, hepatitis C virus (IPR002166) and the third ORF resulted in a detailed signature match of the Nodavirus capsid (IPR024292). Phylogenetic analysis of the two predicted RdRp sequences of the second ORF showed that our sequences cluster to the YTLV1 (YP_009553261) and the Wenzhou tombus-like virus 11 (YP_009342051). Based on this, we classified our near-complete viruses as tombus-like (Figure 3B).

Two pools of the *Ae. cantans* mosquitoes, collected early and late in the summer, had many viral hits corresponding to the unclassified RNA virus Wuhan fly virus 4 (WFV4) (Table 2). From one of these pools, we retrieved an 8955 nt long contig. BLASTn analysis of the sequence yielded the Carfax virus (MIN167498) as the top hit, with 68.19% nt identity; however, the query cover was only 8%. The second top hit was the WFV4 (KX883891), with 65.79% nt identity, and similar to the Carfax virus, the query cover was only 10%. The low query cover indicates that our sequence is probably highly divergent from any other in the database. Further, analysing our sequence with NCBI’s ORF finder revealed one ORF coding for a protein of 2744 aa. A BLASTp search of the aa sequence displayed the polyprotein of the Carfax virus (QED21528) as the top hit with 34.59% aa identity and 92% query cover. The hypothetical protein of WFV4 (YP_009342337) was the third top hit, with 34.14% aa identity and 96% query cover. Analysing our polyprotein with InterProScan showed detailed signature matches of a calicivirus coat protein (IPR004005), Picornavirus capsid (IPR001676), dicistrovirus capsid-polyprotein C-terminal (IPR014872), helicase superfamily 3 single-stranded RNA virus (IPR014759), peptidase S1 PA clan (IPR009003) and a RdRp C-terminal domain (IPR001205), in the N- to C-terminal direction. Based on the detailed signature matches of the polyprotein, this virus follows many of the hallmarks of the order *Picornavirales* [33]. The phylogenetic analysis of the polyprotein showed that our sequence clusters with viruses, such as, the Carfax virus (QED21528) and Kinkell virus (AMO03216) in the viral order *Picornavirales*. Therefore, the near-complete virus was classified as a picorna-like virus and due to the high divergence, we named it Flen picorna-like virus after the location of collection (Figure 3C).

### 3.5. Negative-Sense RNA Viruses

The negative-sense RNA viruses observed in our mosquitoes belonged to the viral families/orders *Orthomyxoviridae*, *Peribunyaviridae, Phasmaviridae, Mononegavirales, Articulavirales,* and *Bunyavirales* and represented between 0.1–39% of the viral reads in the mosquitoes pools. Two of the negative-sense RNA viruses detected were further characterized and phylogenetically analysed and fell within the viral orders *Bunyavirales* and *Articulavirales.*

**Figure 4:**
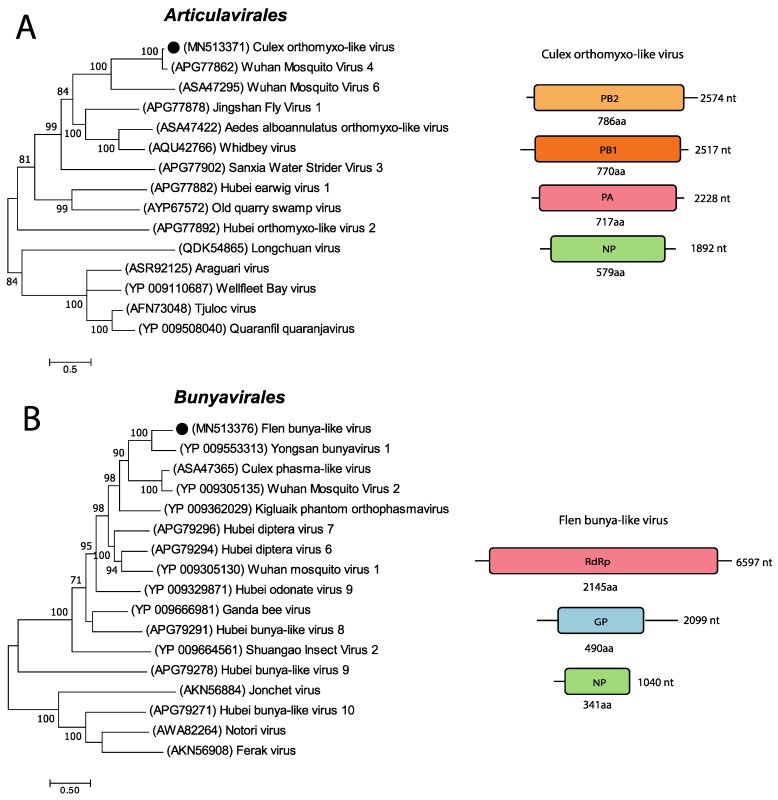
Phylogenetic analysis of the predicted and genomic features of the negative-sense RNA viruses discovered in this study. The maximum-likelihood phylogenetic trees show the positions of newly discovered viruses (solid black circles) in the context of representatives of their closest relatives. The genome structures of the viruses discovered in this study are shown next to their corresponding phylogenies. (**A**) The phylogenetic tree was generated using the translation of the ORF of the PA segment. (**B**) The phylogenetic tree was generated using the translation of the ORF of the large segment.

A partial genome similar to the Wuhan mosquito virus 4 (WMV4) was detected in the *Cx. torrentium* mosquitoes (Figure 4A). WMV4 is an unclassified *Quaranjavirus* with a single-stranded, negative-sense segmented RNA genome. To date, only four segments of generally six segments have been identified, coding for the replicative polymerase complex PB1, PB2, and PA and for the nucleocapsid protein (NP), respectively [7,34]. The length of the segments detected in this study were 2517 nt (PB1), 2574 nt (PB2), 2228 nt (PA), and 1892 nt (NP). BLASTn analysis of the four segments showed 86.86% nt identity to the PB1, 84.30% nt identity to the PB2, 84.92% to the PA and 81.6% nt identity to the NP of WMV4. Analysing each segment with NCBI’s ORF finder, revealed one ORF of 770 aa (PB1), one ORF of 786 aa (PB2), one ORF of 717 aa (PA), and one ORF of 579 aa for segment NP. BLASTp analysis of each ORF showed that the proteins had a high aa similarity to those of WMV4: 95.58% for PB1, 91.22% for PB2, 93.31% for PA and 82.21% for NP. Phylogenetic analysis of the ORF’s aa sequence of the PA segment showed that our sequence clusters with the unclassified *Quaranjaviruses* Wuhan mosquito virus 4 and 6 (KX883866 and MF176380). Based on the characterization of each segment and the phylogenetic analysis, our partial genome was classified as orthomyxo-like and named the Culex orthomyxo-like virus after the mosquito genera it was found in (Figure 4A). 

In the *Ae. cantans* mosquitoes collected early in the summer in Flen, we identified near-full-length genome segments with high similarity to the Yongsan bunyavirus 1 (YBV1). The YBV1 is an unclassified bunyavirus with a linear single-stranded, negative-sense genome divided into three segments [32]. The segments are named small, medium, and large, where the small segment codes for the nucleocapsid protein (NP), the medium segment codes for the glycoprotein (GP), and the large segment codes for the RdRp. The retrieved near-complete genome segments from this study were 1040 nt (small), 2095 nt (medium), and 6596 nt (large) (Figure 4B). BLASTn analysis of each segment, showed a 77.46% nt identity to the NP segment of YBV1, but only with a query cover of 6%. The medium segment showed 66.80% nt identity to the GP segment of YBV1, with a query cover of 35% and the large segment showed 66.37% nt identity to the RdRp segment of YBV1, with a query cover of 59%. Analysing each segment with NCBI’s ORF finder showed one ORF of 341 aa (small segment) with no stop codon, one ORF of 490 aa (medium segment), and one ORF of 2145 aa for the large segment. A BLASTp search of each segment revealed 42.77% aa identity to the YBV1 NP, 55.19% aa identity to the YBV1 GP, and 53.34% aa identity to the YBV1 RdRp. The phylogenetic analysis of the aa sequence of the protein-coding region of the large segment showed that our sequence cluster with the unclassified Bunyavirales YBV1 (YP_009553313), the Orthophasmavirus Wuhan mosquito virus 2 (YP_009305135), and the unclassified virus Culex phasma-like virus (ASA4765) (Figure 4B). Based on the characterization of each segment and the phylogenetic analysis, we classified our near-complete genome as bunya-like and named it Flen bunya-like virus. 

### 3.6. Unclassified Viruses

**Figure 5:**
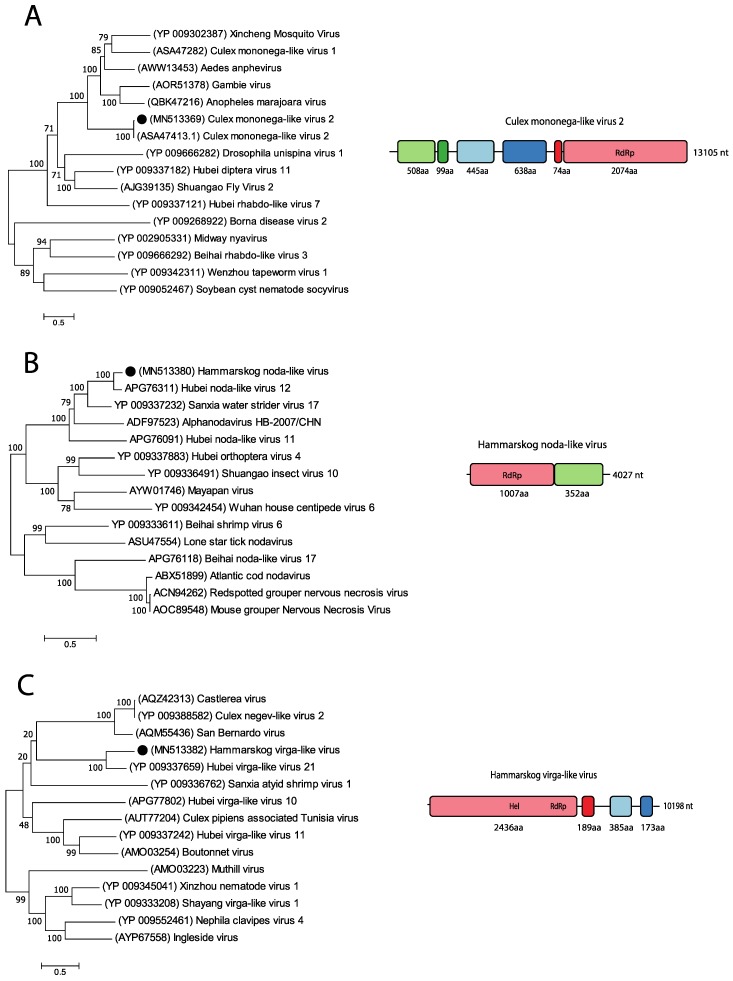
Phylogenetic analysis and genomic features of the unclassified RNA viruses discovered in this study. The maximum-likelihood phylogenetic trees show the positions of newly discovered viruses (solid black circles) in the context of representatives of their closest relatives. The genome structures of viruses discovered in this study are shown next to their corresponding phylogenies. (**A**) The phylogenetic tree was generated using the translation of the sixth ORF. (**B**) The phylogenetic tree was generated using the translation of the first ORF. (**C**) The phylogenetic tree was generated using the translation of the first ORF.

A large proportion of all viral reads were labelled as unclassified RNA viruses (Figure 2A). By investigating theses viral reads in greater detail, we detected the near-complete genomes of three unclassified RNA viruses. Almost all pools had viral reads that mapped to the Culex mononega-like virus 2 (CMLV2), and by assembling overlapping contigs, a near-complete genome of 13,105 nt was identified in the *Cx. torrentium* mosquitoes (Figure 5A). BLASTn of the consensus sequence showed that it had a high nt identity (95.76–96%) to six different strains of CMLV2 (MF176318, MF176377, MF176247, MF176332, MF176268, and MF176298). The top hit (MF176318) has a linear RNA genome of 13,277 nt that codes for six hypothetical proteins. Analysing our CMLV2 sequence with NCBI’s ORF finder software showed similar ORFs coding for similar proteins. Starting from the 5’ end, the first ORF codes for a 508 aa-long protein, BLASTp showed 96.65% identity to a hypothetical protein of CMLV2 (YP_009388617). The second ORF codes for a small protein of 99 aa that shows 98.99% identity to a hypothetical CMLV2 protein (YP_009388618). Analysing the sequence with Phobius showed that it is probably a transmembrane signaling peptide. The third ORF codes for a 445 aa protein with 95.51% nt identity to a hypothetical protein of CMLV2 (ASA47289,). The fourth ORF codes for a protein of 638 aa, BLASTp analysis showed high aa identity 97.96% to the glycoprotein of CMLV2 (YP_009388620). The fifth ORF codes for a small protein of 74 aa, with an aa identity of 90% to a hypothetical protein (ASA47321) of CMLV2. Analysing the sequence in Phobius predicted a transmembrane domain. The last and sixth ORF codes for a long protein of 2074 aa with no stop codon, suggesting that our sequence is not complete. BLASTp analysis showed that the protein has a high aa identity of 96% to the RdRp (ASA47413) of CMLV2. InterProScan revealed detailed signature matches to *Mononegavirales* RdRp (IPR014023), *Mononegavirales* mRNA-capping domain V (IPR026890), and mononegavirus L protein 2-O-ribose methyltransferase (IPR025786). Phylogenetic analysis of the 2074 aa sequence containing the RdRp gene showed the closest association to the CMLV2 (ASA47413), which is an unclassified virus. Two more distant associations were to the unclassified Anphevirus, Anopheles marajoara virus (QBK47216) and the drosophilid anphevirus, Drosophila unispina virus 1 (YP_009666282). As our near-complete genome showed such close genetic identity to other strains of CMLV2 (96% nt identity) [35], we consider this a genotype of CMLV2 (Figure 5A). 

All pools of *Cq. richardii* mosquitoes had viral reads mapping to the unclassified RNA virus Hubei noda-like virus 12 (HNLV 12). However, most of the reads were found in the pool of *Cq. richardii* mosquitoes collected late in the season in Uppsala (Table 2). From this pool, a near-complete genome of 4027 nt was retrieved and BLASTn analysis showed that the sequence had 75.98% nt identity to the HNLV 12 (KX883125). This virus has a linear RNA genome of 4727 nt coding for two hypothetical proteins [5]. Analysing our sequence with NCBI’s ORF finder also revealed two ORFs. The first ORF codes for a protein of 1007 aa, BLASTp search of the aa sequence showed 81% aa identity to the hypothetical protein 1 (APG76311) of Hubei noda-like virus 12. InterProScan showed a signature of a DNA/RNA polymerase (SSF56672). The second ORF codes for a 352 aa protein, with 88.92% aa identity to the hypothetical protein 2 (APG76312) of HNLV 12 and no function could be predicted. Phylogenetic analysis of the 1007 aa sequence, containing the DNA/RNA polymerase, showed that our sequence clusters with viruses in the realm Riboviria, such as the HNLV 12 (APG76311) and the Sanixia water strider virus 17 (YP_009337232). A more distant association in the cluster showed the virus Alphanodavirus HB-2007/CHN (ADF97523), which is an unclassified Alphanodavirus. Our near-complete genome was classified in the realm Riboviria and named Hammarskog noda-like virus based on the characterisation and phylogenetic analysis [36] (Figure 5B). 

In the *Cx. pipiens* pool, a sequence of 10 200 nt was detected with 65.63% nt identity to the Hubei virga-like virus 21 (HVLV 21) (KX883775.1). The HVLV 21 has a linear RNA genome of 10,072 nt, with four ORFs coding for one larger hypothetical polyprotein and three smaller hypothetical proteins [5]. Analysing our sequence with NCBI’s ORF finder showed four ORFs with similarity to HVLV 21 (KX883775). The first ORF codes for the large protein of 2436 aa, with 47.20% aa identity to HVLV 21 (YP_009337659), the second ORF codes for a 189 aa protein with 48.15% aa identity to HVLV 21 (YP_009337660), the third ORF codes for a protein of 385 aa with 36.57% aa identity to HVLV 21 (YP_009337661) and the fourth ORF code for a 173 aa protein with 45.56% aa identity to HVLV 21 (YP_009337662). Analysing the aa sequences with InterProScan predicted that the protein of the first ORF could possess an Alphavirus-like methyltransferase (MT) domain (IPR002588), (+)RNA virus helicase core domain (IPR027351), Tymovirus, RdRp (IPR001788), and a RdRp, catalytic domain (IPR007094). No function or domain could be predicted for the other ORFs. Phylogenetic analysis of the aa sequence of the first ORF showed that our sequence only clusters with the HVLV 21 (YP_009337659). More distant associations were to the unclassified viruses Boutonnet virus (AMO03254) and Sanxia atyid shrimp virus 1 (YP_009336762). Our near-complete genome was classified in the realm Riboviria and named Hammarskog virga-like virus based on the characterisation and phylogenetic analysis [37] (Figure 5C).

## 4. Discussion

We have used a viral metagenomic approach to characterize the virome of mosquitoes collected at two geographic locations in Mid-Eastern Sweden representing six mosquito species. A homology search of the viral reads showed that most of the mosquito viromes were highly diverse, representing multiple viral families (Table 2, Figure 2). Comparing viral hits across the mosquito species and genera, some similarities could be observed, such as viral hits to the CMLV2 and Zhee mosquito virus in almost all pools (Table 2). We could also observe similarities within mosquito genera, such as viral hits to the Culex Iflavi-like virus 3 in the *Culex* mosquitoes and viral hits to the Yongsan bunyavirus 1 in the *Aedes* mosquitoes. Further, many viral hits could only be observed in one of the mosquito species, indicating a host restriction. One example is that only the *Coquillettidia* mosquitoes had viral reads that mapped to the YPLV3 (Table 2). Overall, from our data, we can observe indications that the *Aedes* genera seem to harbour a more diverse negative-sense RNA virome compared to the *Culex* and *Coquillettidia* genera. The *Coquillettidia* mosquitoes had more viral hits among the unclassified RNA viruses compared to the others and the *Culex* mosquitoes had few but very abundant viruses, one that represented more than half of the total viral reads in the *Cx. pipiens* pool (Table 2). However, the mosquito sampling was biased between the species collected and the location, e.g., 350/433 of the *Cq. richardii* were collected in Uppsala and 371/407 of the *Ae. cantans* were collected in Flen (Figure 1). If this reflects the species present at the different locations, the environment of the locations or the different traps used in Flen and Uppsala is hard to say. To draw any conclusions regarding local differences of the viromes, we would need a larger sampling, covering more geographic regions of Sweden and bigger sampling size of each mosquito species from each location. Neither have the different sequence depth and number of mosquitoes in each pool been accounted for in this comparison.

The near-complete genomes of nine RNA viruses were characterized and phylogenetically analysed in greater detail. Four of these viruses were associated to (+)ssRNA viruses in the orders *Picornavirales* and *Tymovirales*. Two viruses were associated to (-)ssRNA viruses in the orders *Bunyavirales* and *Articulavirales* and three viruses could not be classified to an order and have instead been classified in the realm Riboviria, which is the basal rank for RNA viruses [38]. Phylogenetic analyses of these nine viruses showed the closest association to viruses previously detected in mosquitoes or other invertebrates (Figure 3, Figure 4 and Figure 5). This could indicate that these viruses are potentially insect-specific, however, to confirm host specificity, we would need to perform infection studies in different animals or vertebrate cell-lines. Many of the viral reads in our study, especially those unclassified, showed similarity to viruses identified in a metagenomic study from China, by Shi et al. [5], that investigated 220 invertebrate species and discovered 1445 novel viruses. Moreover, four of the near-complete viruses detected in this study were closely associated to the Yongsan picorna-like virus 3, Yongsan bunyavirus 1, and Yongsan tombus-like virus 1. These viruses were found in metagenomic studies of mosquitoes collected at the Yongsan U.S Army Garrison, in the Republic of Korea [32,39]. Further, the CMLV2 detected in this study showed close genetic identity (95.76–96% nt identity) to the CMLV2 discovered in mosquitoes collected in Australia [20]. The majority of viral sequences that our viral reads and near-complete genomes mapped to, have been discovered in mosquitoes far from Northern Europe and Sweden. Providing reference genomes from wider geographic regions and different mosquito species will facilitate future in silico recognition and assembly of viral genomes in metagenomic datasets. 

Two of the nine characterized viruses in this study showed association to a viral family related to plant viruses. The FTLV and HTLV were closely associated to the *Tombusviridae* virus YTLV1 (NC_040725), with 67.63% and 75.17% nt identity. The family *Tombusviridae* is currently associated with plant viruses [40]. Assigning host tropism in metagenomic analysis of whole mosquitoes is hard, as it may include plant viruses from their diet and viruses of parasitic or commensal organisms residing inside or on the mosquito. However, when mapping the reads from respective pools to the FTLV or HTLV sequences, 27,318 reads mapped to HTLV and 48,980 reads mapped to FTLV. This could indicate high abundance of the virus in the mosquito and possibly replication. Further work is needed to confirm replication of these two viruses in mosquito cells. 

This study expands the knowledge of viruses circulating in different mosquito species in Northern Europe. It provides information on the diversity of the viromes of the respective mosquito species investigated, as well as shows the presence of a number of viruses not known to circulate in this region and that are highly divergent to previously characterized viruses. Whether any of these viruses have any direct or indirect potential veterinary or public health-relevance remains to be further investigated. 

## Figures and Tables

**Table 1:** Summary of the mosquito pools that were sequenced. Mix of sample location means that mosquitoes in this pool were collected both in Flen and Uppsala. Mix of sample time point means that mosquitoes in this pool were collected over several time points. *Culex* (*Cx*).

Pool Name	Mosquito spp	Sample Location	Sample Time Point	Raw Reads	Diptera Reads (%)	Microbial Reads (%)	Viral Reads (%)
1	*Cx. pipiens*	Mix	Mix	2 985 629	7.6%	27.55%	8.76%
2	*Cx. torrentium*	Mix	Mix	4 713 173	42.65%	56.21%	4.86%
3	*Ae. commnus*	Mix	Mix	8 590 873	1.43%	13.78%	1.44%
4	*Ae. annulipes*	Mix	Mix	1 842 681	5.42%	27.65%	12.47%
5	*Ae. cantans*	Flen	May and June	1 427 446	5.35%	24.75%	11.00%
6	*Ae. cantans*	Flen	July	5 877 722	3.3%	13.23%	5.43%
7	*Ae. cantans*	Flen	August	2 269 961	5.11%	19.73%	8.48%
8	*Ae. cantans*	Uppsala	mix	1 238 350	6.5%	23.54%	8.00%
9	*Cq. richardii*	Uppsala	June	5 201 595	14.02%	26.8%	3.13%
10	*Cq. richardii*	Uppsala	July	8 889 584	12.6%	26.37%	5.39%
11	*Cq. richardii*	Uppsala	August	4 821 295	9.19%	27.73%	10.4%
12	*Cq. richardii*	Flen	Mix	4 247 080	12.2%	29.19%	5.81%

**Table 2:** Viral hits from the homology search of high-quality reads for each pool. Viral hits are shown as percentage of total reads for each mosquito pool, the different pools are described in Table 1. Viruses with less than 4000 mapped reads in any of the pools were aggregated under other viral reads.

Abundance of Viruses (% of Total Reads)												
	Cx. Pipiens	Cx. Torrentium	Ae. Communis	Ae. Annulipes	Ae. Cantans				Cq. Richardii			
**Viruses**	1	2	3	4	5	6	7	8	9	10	11	12
												
**Negative-sense ssRNA virus**												
Wuhan Mosquito Virus 4	**0.009**	**1.897**	0	0	0	0	0	0	**0.166**	**0.098**	**0.044**	**0.073**
Wuhan Mosquito Virus 3	0	0	0	0	**0.031**	**0.01**	**0.119**	**0.006**	0	0	0	0
Xinzhou Mosquito Virus	0	0	**0.058**	**0.018**	**0.004**	0	**0.006**	0	**0.026**	**0.042**	**0.053**	**0.17**
Zhee Mosquito virus	0	0	**0.043**	**0.137**	**0.066**	**0.012**	**0.078**	**0.021**	**0.009**	**0.018**	**0.027**	**0.01**
Culex Bunya-like virus	0	0	**0.028**	**0.252**	**0.065**	**0.003**	**0.066**	0	**0.095**	**0.432**	**0.669**	**0.125**
Yongsan bunyavirus 1	0	0	**0.016**	**2.844**	**0.852**	**0.169**	**0.177**	**2.235**	0	0	0	0
Xincheng anphevirus	0	0	**0.004**	**0.142**	**0.014**	**0.003**	0	**0.025**	0	0	0	0
Anopheles darlingi virus	0	0	**0.003**	**0.381**	**0.005**	**0.001**	0	**0.009**	0	0	0	0
Wuhan mosquito 2	0	0	**0.003**	**0.043**	**0.215**	**0.099**	**0.122**	**0.022**	0	0	0	0
Whidbey virus	0	0	0	0	**0.042**	**0.009**	**0.102**	**0.007**	0	0	0	0
												
**Positive-sense ssRNA virus**												
Yongsan tombus-like virus 1	0	0	0	**1.511**	**2.142**	**0.02**	**0.835**	**0.027**	**0.269**	0	**0.002**	0
Yongsan picorna-like virus 3	0	0	0	0	0	0	0	0	**0.124**	**0.297**	**1.279**	**0.002**
Yongsan sobemo-like virus 1	0	0	**0.015**	0	0	0	0	0	**0.009**	**0.011**	**0.004**	**0.021**
Culex Iflavi-like virus 3	**0.003**	**2.298**	0	0	0	0	0	0	0	0	0	0
Kinkell virus	0	0	0	0	**0.087**	0	**0.109**	0	0	0	0	0
Mosquito nodavirus MNV-1	0	0	0	**0.006**	**0.007**	0	**0.003**	0	**0.042**	0	0	0
Alphanodavirus HB-2007/CHN	0	0	0	0	0	0	0	0	0	**0.025**	**0.026**	0
												
**Unclassified RNA virus**												
Hubei noda-like virus 6	0	0	0	0	**0.035**	**0.034**	0	**0.042**	0	**0.002**	**0.002**	0
Hubei virga-like virus 21	**4.916**	0	0	0	0	0	0	0	0	0	0	0
Hubei noda-like virus 12	0	0	0	0	0	0	0	0	**0.023**	**1.197**	**4.219**	**0.045**
Hubei partiti-like virus 22	0	0	0	**0.112**	0	**2.501**	**0.009**	**0.007**	0	0	0	0
Hubei diptera virus 17	0	0	0	0	0	0	**0.001**	0	**0.054**	**0.028**	**0.025**	**0.076**
Hubei diptera virus 13	0	0	0	0	0	0	0	0	**0.025**	**0.022**	**0.013**	**0.074**
Hubei sobemo-like virus 8	0	0	0	0	0	0	0	0	**0.04**	**0.047**	**0.02**	**0.064**
Hubei sobemo-like virus 9	0	0	0	0	0	0	0	0	**0.034**	**0.041**	**0.022**	**0.069**
Hubei mosquito virus 2	0	0	**0.075**	0	0	0	0	0	0	0	0	0
Hubei tetragnatha maxillosa virus 8	0	0	0	0	0	0	0	0	**0.015**	**0.054**	**0.054**	**0.48**
Wuhan insect virus 13	0	**0.29**	0	0	0	0	0	0	0	0	0	0
Wuhan fly virus 4	0	0	0	0	**0.612**	0	**1.05**	0	0	0	**0.001**	**0.003**
Wenzhou sobemo-like virus 4	0	0	**0.034**	0	0	0	0	0	0	0	0	0
Sanxia water strider virus 17	0	0	0	0	0	0	0	0	0	**0.035**	**0.052**	0
Shuangao insect virus 12	0	0	0	0	**0.024**	0	**0.166**	0	0	0	0	0
Renna virus	0	0	**0.038**	0	0	0	**0.001**	0	0	0	0	0
Culex mononega-like virus 2	0	**0.216**	**0.112**	**0.721**	**0.091**	**0.017**	0	**0.189**	**0.005**	**0.004**	**0.004**	**0.002**
Culex mononega-like virus 1	0	0	**0.006**	**0.112**	**0.012**	**0.003**	0	**0.018**	0	0	0	0
Ae camptorhynchus negev-like virus	0	0	0	0	**0.359**	**0.11**	**0.654**	**1.902**	0	0	0	0
Ae alboannulatus orthomyxo-like virus	0	0	0	0	**0.183**	**0.038**	**0.323**	**0.015**	0	0	**0.002**	0
Ae camptorhynchus reo-like virus	0	0	**0.046**	0	0	0	0	0	0	0	0	0
Salarivirus Mos8CM0	0	0	**0.018**	**0.707**	**0.597**	**0.19**	**0.819**	**0.326**	**0.01**	**0.009**	**0.015**	**0.04**
Chaq virus-like 1	0	0	0	0	0	0	0	0	**0.126**	**0.032**	**0.025**	**0.366**
uncultured virus	0	0	**0.021**	**0.032**	**0.128**	**0.015**	**0.071**	**0.021**	**0.251**	**0.347**	**0.434**	**0.485**
Caninovirus sp.	0	0	0	0	**0.068**	**0.082**	0	**0.096**	0	0	0	0
												
**Total reads in table (%)**	**4.93**	**4.7**	**0.52**	**7.02**	**5.64**	**3.32**	**4.71**	**4.97**	**1.32**	**2.74**	**7**	**2.11**
**Other viral reads (%)**	**3.83**	**0.16**	**0.92**	**5.45**	**5.36**	**2.11**	**3.77**	**3.03**	**1.81**	**2.66**	**3.4**	**3.7**
**Total viral reads (%)**	**8.76**	**4.86**	**1.44**	**12.47**	**11**	**5.43**	**8.48**	**8**	**3.13**	**5.4**	**10.4**	**5.81**

**Table 3:** Summary of the BLASTp analysis of each ORF of FTLV and HTLV.

ORFs	FTLV Protein	HTLV Protein	aa Identity between FTLV and HTLV	aa Identity to YPLV1 (FTLV)	aa Identity to YPLV1 (HTLV)	Accession Number
ORF 1	411 aa	379 aa	46.88%	43.98%	70.59%	YP_009553260
ORF 2	479 aa	482 aa	65.13%	66.6%	81.17%	YP_009553261
ORF 3	412 aa	409 aa	62.77%	64.63%	87.5%	YP_009553263
